# Structural Characterization and Antioxidant Activity of Milled Wood Lignin from Xylose Residue and Corncob

**DOI:** 10.3390/polym11122092

**Published:** 2019-12-13

**Authors:** Miaomiao Xu, Chao Wang, Gaojin Lyu, Lei Zhong, Liyuan Yang, Zhiwei Wang, Chengrong Qin, Xingxiang Ji, Guihua Yang, Jiachuan Chen, Feng Xu

**Affiliations:** 1 State Key Laboratory of Biobased Material and Green Papermaking, Qilu University of Technology, Shandong Academy of Sciences, Jinan, Shandong 250353, China; 2 Guangxi Key Laboratory of Clean Pulp & Papermaking and Pollution Control, Guangxi University, Nanning, Guangxi 530004, China; 3 Beijing Key Laboratory of Lignocellulosic Chemistry/MOE Key Laboratory of Wooden Material Science and Application, Beijing Forestry University, Beijing 100083, China

**Keywords:** lignin, characterization, antioxidant activity, corncob, HSQC

## Abstract

Xylose residue (XR), after diluted acid treatment of corncob, consists of cellulose and lignin. However, structural changes of XR lignin have not been investigated comprehensively, and this has seriously hindered the efficient utilization of lignin. In this study, corncob milled wood lignin (CC MWL), and xylose residue milled wood lignin (XR MWL) were isolated according to the modified milled wood lignin (MWL) method. The structural features of two lignin fractions were thoroughly investigated via fourier transform infrared spectroscopy (FTIR), gel permeation chromatography (GPC), thermogravimetric analysis (TGA) and two dimensional nuclear magnetic resonance (2D NMR) spectroscopy techniques. XR MWL with higher yield and lower bound carbohydrate contents presented more phenolic OH contents than CC MWL due to partial cleavage of *β*-O-4. Furthermore, the molecular weights of XR MWL were increased, possibly because of condensation of the lignin during the xylose production. A study on antioxidant activity showed that XR lignin had better radical scavenging ability than that of 2,6-Di-tert-butyl-4-methyl-phenol (BHT) and CC MWL. The results suggested that the lignin in xylose residue, showing great antioxidant properties, has potential applications in food additives.

## 1. Introduction

Efficient exploitation of lignocellulose for producing bio-fuels, bio-chemicals, and bio-materials can decrease the consumption of fossil fuels [1]. Xylose residue is a kind of waste biomass during the xylitol manufacture from corncobs [2,3]. Xylose production is the first step of corncob utilization by means of diluted acid treatment. To further improve the economic value, the xylose residue (XR) can potentially applied to the bioethanol production by bioconversion processes [4]. However, the presence of a large amount of lignin still significantly reduced the efficiency of the enzymatic hydrolysis [5]. With the aim to reveal the hindrance of residual lignin during the subsequent enzymatic hydrolysis/fermentation, it is necessary to investigate the structural variation of lignin during the production of xylose. On the other hand, lignin with many functional groups, can be used for the production of biomaterials, such as carbon fibers, phenolic resins, and other high value-added chemicals. Accordingly, the efficient utilization of lignin in XR was another approach to improve the economic benefits of xylose and bioethanol [6,7]. However, the comparable structure alterations of lignin in different biorefineries have not been clearly understood due to the different raw feeds and chemical technology. Hence, it is essential that the comparative characterizations of the lignin in the corncob biorefineries, prior to utilization of lignin for chemicals and materials, facilitated the further development of bio-chemicals and bio-materials production.

In this work, a modified milled wood lignin (MWL) preparation was successively isolated from two feeds (corncob and xylose residue). All kinds of systematic techniques containing fourier transform infrared spectroscopy (FTIR), gel permeation chromatography (GPC), thermogravimetric analysis (TGA), quantitative ^31^P nuclear magnetic resonance (^31^P NMR) spectroscopy and two dimensional heteronuclear single quantum coherence nuclear magnetic resonance (2D-HSQC NMR) spectroscopy, determined structural properties of two lignin samples. Furthermore, the antioxidant activity was investigated, because of the close relation between the typical structure and possible application. Comparative characterizations of lignin form reaction stages in the corncob biorefinery will not only facilitate more effective deconstruction of lignocellulose, but also enable faster development of bio-fuel, bio-chemicals, and bio-materials production from byproduct lignin.

## 2. Materials and Methods 

### 2.1. Materials

The xylose residue and corncob were obtained from Shandong Longlive Corporation (Dezhou, Shandong, China). The samples were firstly washed several times by distilled water, and then dried at 55 °C overnight. The dried samples were milled to a size under 40 meshes. Then the two samples were extracted with a 2/1 (v/v) toluene/ethanol mixture for 8 h. Chemical components of the extractive-free materials were analyzed by employing the national renewable energy laboratory (NREL) procedure [8]. 

### 2.2. Isolation of MWLs from the Two Feedstocks

The two MWL fractions were separated according to the procedure of MWL isolation [9,10]. The extractive-free samples (100 g) were finely ball-milled in ZrO_2_ vessels (500 mL) by a planetary ball mill (Fritsch, Germany). Each sample was ground for 5 h with 10 min breaks between 10 min working. To prepare the MWL, the milled sample was extracted by 96/4 dioxane/H_2_O mixture for 48 h, and then centrifuged. The dioxane was distilled off under reduced pressure. The crude lignin was precipitated in 3 volumes of deionized water. The crude lignin was washed by the 0.01 M HCl solution, and lyophilized. The relatively pure lignin fractions were dissolved in 2/1 (v/v) dichloromethane/ethanol. The insoluble precipitate was washed with diethyl ether, and then centrifuged, and finally freeze-dried to obtain purified lignin [11].

### 2.3. Characterization of MWLs

The associate carbohydrates were analyzed according the typical procedure of NREL [8]. FTIR spectra of lignin samples was acquired by a Nicolet iN 10 infrared spectrometer (Thermo, Waltham, MA, USA). The scan times were set to 64. The GPC analysis of MWLs were conducted using Agilent 1260 gel permeation chromatography (Agilent, Santa Clara, CA, USA). The analytical column in this study was a PL-gel Mixed-B type. Before injecting the samples, the 10 mg lignin fractions were firstly dissolved in 20 mL THF. The column temperature was set to 30 °C. Polystyrene standards are used to calibrate the column. NMR spectra was collected on an AVIII 400 MHz instrument (Brucker, Germany). For the ^31^P NMR experiment, 20 mg MWLs were dissolved in 1.6/1 pyridine/deuterated chloroform with a total volume of 500 μL. The cyclohexanol was the internal standard. Before NMR analysis, the mixture was reacted with 100 μL 2-chloro-1,3,2-dioxaphospholane [12]. For the HSQC experiment, the signal of the dimethylsulfoxide (DMSO) peak was used as an internal reference (39.5 ppm and 2.49 ppm). We dissolved 60 mg MWLs in 500 μL DMSO-d_6_ [13]. Thermal analysis was carried out by Q200 thermal analyzer (TA, New Castle, PA, USA). About 8–10 mg MWLs were heated from 300 K to 1100 K at a heating rate of 10 K/min in N_2_.

### 2.4. Antioxidant Activity against DPPH Radical

The 2,2-diphenyl-1-picrylhydrazyl (DPPH) free radicals inhibiting capacity of MWL was estimated according to the typical protocol [14]. Typically, MWL was dissolved in 0.1 mL 9/1 (v/v) dioxane/H_2_O. The DPPH concentration was 25 mg/mL in the ethanol solution. Then the MWL solution was mixed to 3.9 mL DPPH solution at 25 °C for 0.5 h. The measure of absorbance was conducted at 517 nm. The DPPH radical-inhibiting activity was calculated: DPPH radical-inhibiting activity (%) = (A_0_−A_1_)/A_0_

Where A_0_ is the absorbance of sample without lignin, and A_1_ is the absorbance of lignin sample. The 50% of DPPH radical-inhibitation activity was defined as IC_50_. The radical scavenging index (RSI) was defined as the inverse of IC_50_. The measurement was conducted in triplicate.

## 3. Results and Discussion

### 3.1. Composition Analysis and Associated Carbohydrates

The carbohydrates and lignin in the corncob and xylose residue were presented in Table 1. The lignin content in corncob was 13.3 wt%. As can be seen, glucan and xylan were the major compositions in the corncob. After diluted acid hydrolysis, the lignin relative contents in xylose residue increased to 23.1 wt%. In addition, the glucan contents in the xylose residue sharply increased to 60.2 wt%, while the xylan contents decreased to 5.1 wt% [15]. The total structural composition of xylose residue was slightly lower than that of the original corncob.

Table 2 showed that the isolated yield and residual sugars of MWL fractions. The yields of CC MWL and XR MWL were 5.25% and 8.78%, respectively. Clearly, the two lignins contained low yields of associated carbohydrates. In particular, xylose with relatively much content (1.58 wt%) was in CC MWL. As can be seen, glucose and xylose were two main carbohydrates, and arabinose was still existed in lignin samples. The decrease of xylose content in XR MWL suggested that the ether bonds between xylan and lignin were cleaved after diluted acid pretreatment [16].

### 3.2. Molecular Weight of Lignin Fractions

The values of the weight-average (*Mw*) and number-average (*Mn*), and the polydispersity index (PI: *Mw/Mn*) are shown in Table 3. The two kind of molecular weights of XR MWL were higher than that of CC MWL, possibly because of improved degree of branching and condensation after diluted acid pretreatment. It may be that lignin mostly existed in the residue after acid pretreatment, as it is less exposed during the acid pretreatment process [4,17]. Furthermore, the PI of XR MWL increased during the xylose production process, indicating that the diluted acid treatment not only destroyed the macromolecule of lignin, but also caused the increase of un-uniformity of the molecular weight.

### 3.3. FTIR Spectra

**Figure 1:**
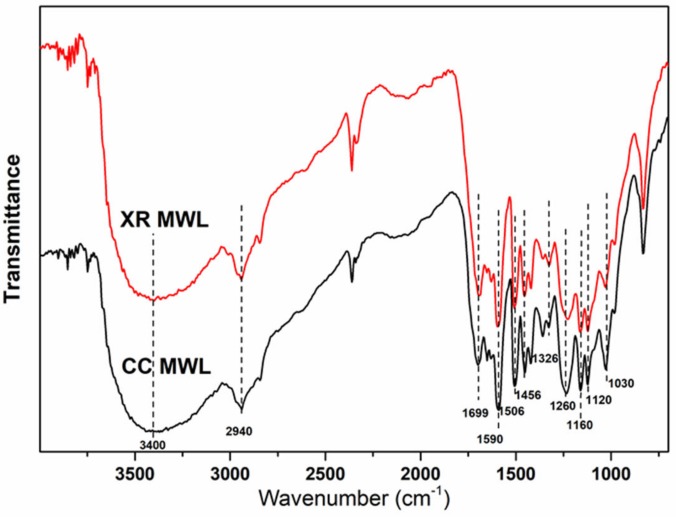
Fourier transform infrared spectroscopy (FTIR) subtraction spectra of the two MWLs.

The change of functional group of two lignin fractions was determined by FTIR according to studies [7,11], and the spectra is shown in Figure 1. The wide peaks at about 3400 cm^−1^ are characterized as the O–H stretch vibration and hydrogen bond in the lignins. Corncob (CC) MWL contained stronger O–H stretching vibrations than XR MWL. The absorption band at 2940 cm^−1^ is originated from the stretch vibration of methyl and methylene in the lignin. C–H stretching of methoxyl groups is present at 2847 cm^−1^. The absorbance band of 1699 cm^−1^ corresponds to unconjugated carbonyl and carboxyl group stretching. The representative bands at 1590, 1506, and 1456 cm^−1^ are attributed to the aryl ring stretching of lignin. We found that the typical structure of lignin did not alter after acid pretreatment processes. The peaks at 1326 cm^−1^ correspond to syringyl and noncondensed guaiacyl ring breathing with a C–O stretch [18]. The band at 1260 cm^−1^ is attributed to guaiacyl ring breathing with a C=O stretch. The peaks at 1160 cm^−1^ in the spectra were observed, which indicates the existence of an ester bond with p-hydroxyphenyl units of grass lignin, indicating the occurrence of an H–typed structure. The 1120 cm^−1^ band corresponds to an aromatic C–H in plane deformation. The intensity of the 1030 cm^−1^ C–O–C band was decreased after acid pretreatment, indicating that a partial ether bond was cleaved during the diluted acid treatment [19].

### 3.4. Quantitative NMR Spectra

#### 3.4.1. ^2^D-HSQC Spectra

**Figure 2:**
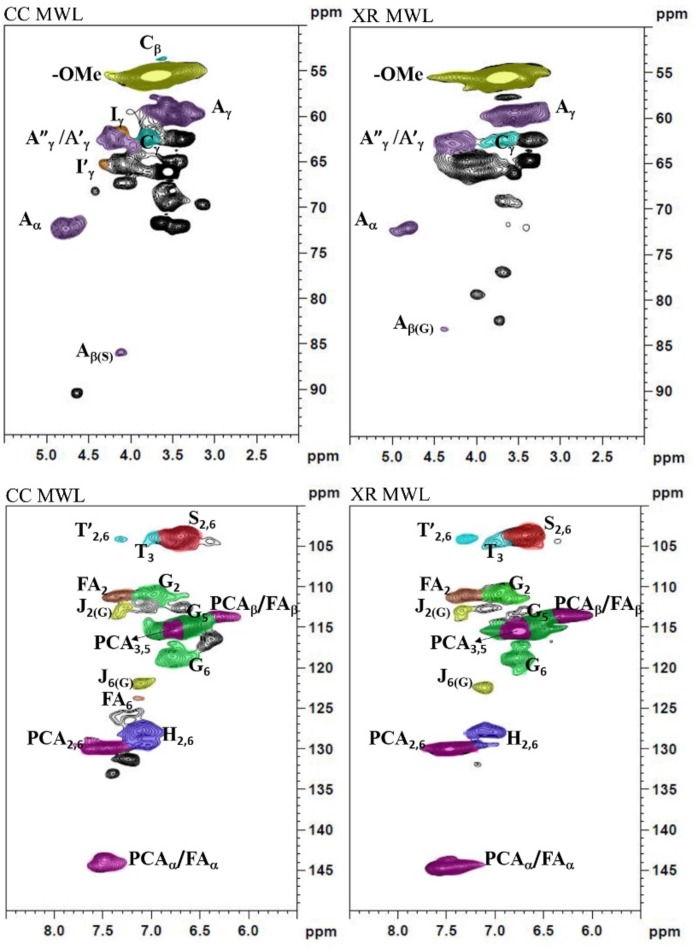
Two dimensional heteronuclear single quantum coherence nuclear magnetic resonance (2D-HSQC NMR) spectra and the main structures of lignin samples.

To study the structural aspects of complex lignin, quantitative ^2^D-HSQC NMR were required for qualitative and quantitative understanding of the lignin structure. The side chain (δ_C_/δ_H_ 50.0–95.0/2.00–5.50) and aromatic regions (δ_C_/δ_H_ 100.0–150.0/5.50–8.50) of the two MWLs are shown in Figure 2.

The lignin isolated from corncob contains a considerable number of β-ether unit (A), with some detectable phenylcoumaran (C) [16]. The signals of methoxyl groups in XR MWL in Figure 2 are smaller than those in CC MWL, indicating that the lignin of corncob was possibly demethoxyled during the dilute acid pretreatment process. The signal at 63.1/4.22 in the HSQC spectrum of MWL corresponds to the Cγ–Hγ position of γ-*p*-counmaroylated β-ether units A (γ-*p*CA) (Figure S1 and Table S1). Furthermore, the signal at 61.3/4.09 corresponds to the Cγ–Hγ position of *p-*hydroxycinnamyl (sinapyl/coniferyl) alcohol. This finding is consistent with the eucalypt MWL [20]. Signals of syringyl (S), guaiacyl (G), and *p*-hydroxyphenyl (H) are clearly distinguished. Except to the signals mentioned above, the correlations of the *p*-coumaric acid (*p*CA) and ferulic acid (FA) are found. Furthermore, the aromatic C_3_−H_3_ (δ_C_/δ_H_ 104.7/7.03) and C_2′,6′_–H_2′,6′_ (δ_C_/δ_H_ 103.9/7.30) signals exist in Figure 2 [21]. During the biorefinery process, the content of tricin was gradually increased. This significant finding is consistent with that in previous studies [22,23].

The calculated S/G ratios were 1.48 and 1.12 for CC MWL and XR MWL, respectively. XR MWL had a lower S/G ratio than that of CC MWL. Lignin fractions in the middle lamella contained relatively more G units; whereas lignin in the secondary wall S2 had relatively more S units. We believe that lignin from xylose residue was easily isolated from the middle lamella after acid treatment. In addition, the β–O–4 of CC MWL (37.10/100Ar) was more than in the XR MWL (9.22/100Ar). The results were attributed to partial cleavage of β*–*O–4 aryl ether after diluted acid pretreatment [17].

#### 3.4.2. ^31^P NMR Analysis of Lignins

**Figure 3:**
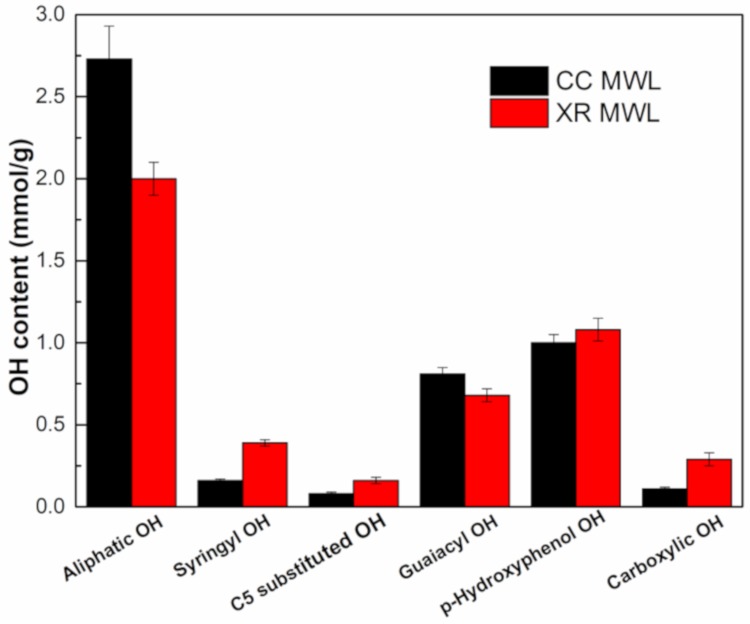
Hydroxyl group contents in CC MWL and XR MWL.

^31^P NMR is regularly used to quantify different hydroxyl groups (aliphatic OH, phenolic OH, and carboxylic acids) in the lignin structure [12]. The integration data of aliphatic and phenolic OH groups for the two MWLs are listed in Figure 3.

After diluted acid pretreatment, the content of aliphatic OH groups decreased from 2.73 mmol/g to 2.0 mmol/g, owing to the cleavage of ether bonds. The content of condensed OH greatly increased from 0.08 mmol/g to 0.16 mmol/g, indicating that some monophenols were condensed during the production of xylose process. Total phenolic OH increased from 2.05 mmol/g to 2.30 mmol/g. Furthermore, the carboxylic group increased significantly from 0.11 mmol/g to 0.29 mmol/g, which may be due to occurrence of an oxidation reaction in the diluted acid pretreatment or the ball milling process [17].

### 3.5. Thermal Analysis

**Figure 4:**
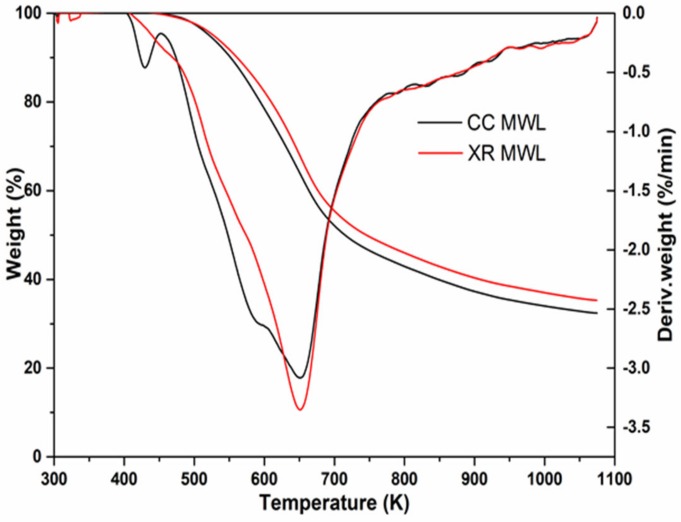
Thermogravimetric analysis (TGA) and differential thermogravimetric (DTG) curves of lignins.

The thermal properties are significant for thermochemical conversion of isolated lignin or raw biomass. The trend of the thermal conversion of CC MWL and XR MWL was similar, but there some differences shown in Figure 4. It was shown that the first stage of weight loss at 300–430 K was owing to the loss of absorbed water. The second stage of weight loss occurred at 575–770 K. The maximum rates of weight loss occurred at 630–655 K. This result was similar to the previous reports [16]. The temperature with the maximum degradation rate of XR MWL was 652 K, higher than that of CC MWL, due to the increase in thermal stability with the increasing molecular weight [13]. From the results reported by a previous worker, the higher ash may have contributed to lowering the peak temperature by effecting the degradation rate by weakening the C–O bond in the β–O–4 unit [24]. The residue yields (char) at the final temperature were 32.0% for CC MWL, and 35.0% for XR MWL. When the sample was MWL from furfural residue (FR) of corncob, the residual weight was 37%. These results indicated that lignin from FR after relatively serious acid pretreatment containing more condensed structures [25]. It was concluded that the lignin isolated from xylose residue had more condensed structures than that from corncob, which was also consistent with the previous GPC and NMR results.

### 3.6. Antioxidant Activity of MWLs

**Figure 5:**
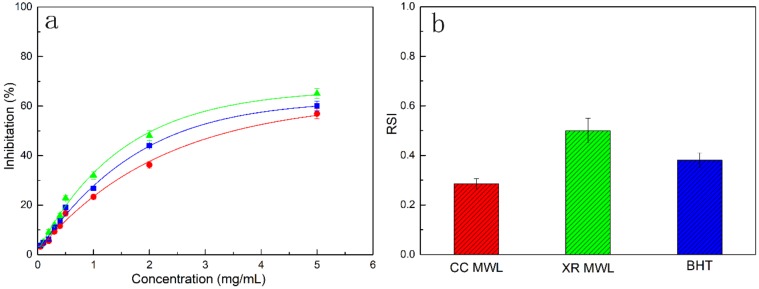
Antioxidant activity against 2,2-diphenyl-1-picrylhydrazyl radicals (DPPH) of CC lignin and XR lignin as compared to 2,6-Di-tert-butyl-4-methyl-phenol (BHT). (**a**) DPPH inhibitory effect; and (**b**) radical scavenging index (RSI) value.

The antioxidant properties of lignins in raw corncob and treated xylose residue are compared to the commercial antioxidant, BHT. The DPPH inhibitory effects of these fractions were shown in Figure 5. Two MWLs exhibited antioxidant activity. As the concentrations increased, the effect against DPPH was improved sharply. The RSI values of CC MWL and XR MWL were 0.28 and 0.49 while the RSI values of BHT was 0.38. The antioxidant activity of CC MWL was lower than BHT, indicating that the CC MWL had less aliphatic hydroxyl groups. Commonly, the capacity of phenolic samples relied on the forming ability of the phenoxyl radical by donating hydrogen atoms. The XR MWL exhibited higher DPPH inhibitory effect than CC MWL. The results indicated that lignin in xylose residue had relatively high antioxidant activity. Hence, the lignin in xylose residue may be a potential antioxidant of food oils and fats for food preservatives [26].

## 4. Conclusions

In conclusion, the structures of CC MWL and XR MWL were studied in detail. In XR MWL, less bound carbohydrate content and relatively high isolation yields were found. In addition, the molecular weight of XR MWL was higher than that of CC MWL, indicating that condensation was the main side reaction during the xylose manufacture. Furthermore, the residual weight of the XR MWL was higher than CC MWL. Compared with CC MWL, XR MWL contains more phenolic hydroxyls and carboxyls, suggesting that cleavage of β–O–4, and carboxyl formation were main structural changes. The XR MWL showed good antioxidant activity, which was higher than that of BHT. This suggested that the lignin in xylose residue could show potential application as a natural antioxidant.

## Figures and Tables

**Table 1:** Chemical composition of corncob (CC) and xylose residue (XR).

Samples	Ara ^a^	Glu ^a^	Xyl ^a^	Gal ^a^	Lignin	Others
CC	5.05	39.14	32.43	2.39	13.31	7.68
XR	0.20	60.22	5.04	-	23.12	11.42

^a^ Ara: Arabinan, Glu: glucan, Xyl: xylan, Gal: galactan.

**Table 2:** Content of neutral sugar of the isolated milled wood lignin (MWL).

Sample	Yield ^a^	Ara ^b^	Glu ^b^	Xyl ^b^	Total Sugar ^b^
CC MWL	5.25	0.21	0.85	1.58	2.64
XR MWL	8.78	0.14	0.50	0.47	1.11

^a^ Based on Klason lignin in sample. ^b^ Ara: Arabinan, Glu: Glucose, Xyl: Xylan.

**Table 3:** Molecular weight and polydispersity index (PI) of the two MWLs.

Sample	*Mw*	*Mn*	PI (*Mw/Mn*)
CC MWL	2847	2283	1.25
XR MWL	6121	4104	1.49

## Supplementary Materials

The following are available online at https://www.mdpi.com/2073-4360/11/12/2092/s1, Figure S1: Main structures of lignin fractions of corncob, involving different side-chain linkages, and aromatic units by 2D HSQC NMR, Table S1: Assignments of main lignin ^13^C-^1^H correlation signals in the HSQC NMR spectra shown in Figure 2.
