# Supplementary material for: Structural Characterization and Antioxidant Activity of Milled Wood Lignin from Xylose Residue and Corncob
Source: Polymers (Basel). 2019 Dec 13;11(12):2092. doi: 10.3390/polym11122092 (PMC6960613; doi:10.3390/polym11122092)
Supplement: Supplementary file 1 [file polymers-11-02092-s001.pdf]

Supporting Information

Structural Characterization and Antioxidant Activity of Milled Wood Lignin from Xylose Residue and Corncob

Miaomiao Xu[1], Chao Wang[1,*], Gaojin Lyu[1,2,*], Lei Zhong[1], Liyuan Yang[1], Zhiwei Wang[2], Chengrong Qin[2], Xingxiang Ji[1], Guihua Yang[1], Jiachuan Chen[1], Feng Xu[1,3]

1 State Key Laboratory of Biobased Material and Green Papermaking, Qilu University of Technology, Shandong Academy of Sciences, Jinan, Shandong 250353, China; xumiaomiaommx@gmail.com (M.X.); zhonglei195@gmail.com (L.Z.); lgjchf@gmail.com (L.Y.); jxx@qlu.edu.cn (X.J.); ygh@qlu.edu.cn (G.Y.); chenjc@qlu.edu.cn (J.C.)

2 Guangxi Key Laboratory of Clean Pulp & Papermaking and Pollution Control, Guangxi University, Nanning, Guangxi 530004, China; wangzhiwei@gxu.edu.cn (Z.W.); qinchengrong@gxu.edu.cn (C.Q.)

3 Beijing Key Laboratory of Lignocellulosic Chemistry/MOE Key Laboratory of Wooden Material Science and Application, Beijing Forestry University, Beijing 100083, China; xfx315@bjfu.edu.cn (F.X.)

* Correspondence: chaowang@qlu.edu.cn (C.W.), Tel: +86-0531-8963-1681 (C.W.); gaojinlv@qlu.edu.cn (G.L.), Tel.: +86-0531-8963-1681 (G.L.)

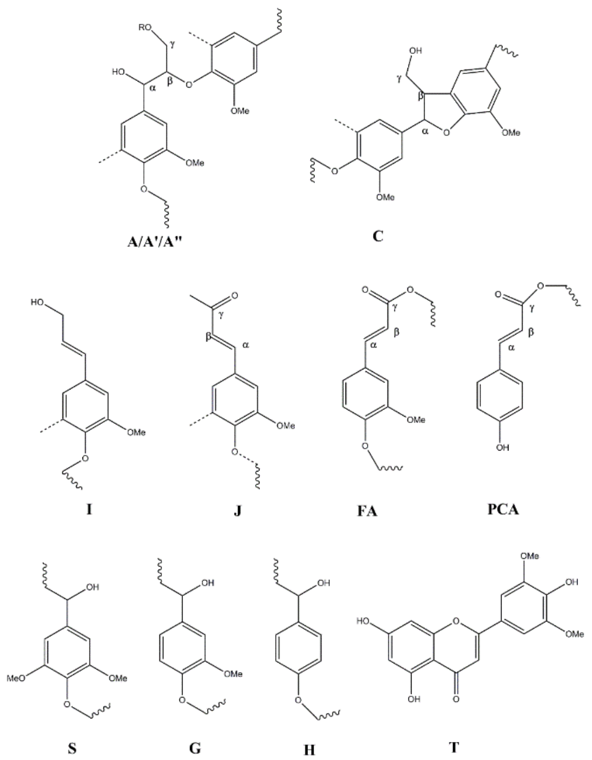

Figure S1. Main structures of lignin fractions of corncob, involving different side-chain linkages, and aromatic units by 2D HSQC NMR. (A) β-O-4′ linkages; (A′) β-O-4 linkages with acetylated g-carbon; (A″) β-O-4 linkages with p-counmaroylated γ-carbon; (C) phenylcoumarance structures formed by β-5′ and α-O-4′ linkages; (I) cinnamyl alcohol end-groups; (J) cinnnamyl aldehyde end-groups; (S) syringyl unit; (G) guaiacyl unit; (H) p-hydroxyphenyl unit; (FA) ferulate; (*p*CA) p-coumarate; (T) tricin.

Table S1. Assignments of main lignin ^{13}C-^{1}H correlation signals in the HSQC NMR spectra shown in Figure 2.

Label	δC/δH	assignment
A_γ	59.65/3.61 and 3.27	C_γ–H_γ in *β*-O-4′ substructures (A)
I_γ	61.3/4.09	C_γ–H_γ in *p*-hydroxycinnamyl (sinapyl/coniferyl) alcohol (I)
A'_γ/A''_γ	62.7/3.83-4.30	C_γ–H_γ in γ-acylated *β*-O-4′ substructures (A′/A″)
I'_γ	64.0/4.79	C_γ-H_γ in γ-acylated cinnamyl alcohol end-groups (I′)
Aα	71.8/4.80	Cα-Cα in *β*-O-4′ substructures
$A_{\beta(G)}$ and $A'_{\beta(S)}$	83.4/4.31	C_β–H_β in *β*-O-4′ substructures linked to a G unit (A) and in *γ*-acylated *β*-O-4′ substructures linked to a S unit (A′)
$A_{\beta(S)}$	85.9/4.12	C_β–H_β in *β*-O-4′ substructures linked to a S unit (erythro) (A)
$S_{2,6}$	103.7/6.71	$C_{2,6}$-$H_{2,6}$ in etherified syringyl units (S)
$T'_{2,6}$	103.9/7.30	$C_{2',6'}$-$H_{2',6'}$ in tricin (T)
T_3	104.7/7.03	C_3-H_3 in tricin (T)
G_2	110.7/6.98	C_2-H_2 in guaiacyl units (G)
FA_2	111.0/7.32	C_2-H_2 in ferulate (FA)
$J_{2(G)}$	112.24/7.25	C_2-H_2 in cinnamyl aldehyde end-groups (J)
pCA_β and FA_β	113.5/6.27	C_β-H_β in p-coumarate (*p*CA) and ferulate (FA)
G_5	114.9/6.72 and 6.94	C_5-H_5 in guaiacyl units (G)
$PCA_{3,5}$	115.5/6.77	$C_{3,5}$-$H_{3,5}$ in p-coumarate (*p*CA)
G_6	118.7/6.77	C_6-H_6 in guaiacyl units (G)
$J_{6(G)}$	122.3/7.10	C_6-H_6 in cinnamyl aldehyde end-groups (J)
$H_{2,6}$	127.8/7.22	$C_{2,6}$-$H_{2,6}$ in p-hydroxyphenyl units (H)
$PCA_{2,6}$	129.9/7.46	$C_{2,6}$-$H_{2,6}$ in *p*-coumarate (*p*CA)
PCA_α and FA_α	144.7/7.45	C_α-H_α in *p*-coumarate (*p*CA) and ferulate (FA)
